# Machine learning and multi-omics technologies for precision cardiovascular medicine: advancing diagnosis, risk prediction, and therapeutic guidance

**DOI:** 10.3389/fcvm.2026.1921962

**Published:** 2026-08-13

**Authors:** Yaqi Dai, Liufang Wu, Hanliangqi Xue, Liru Zhou, Yahan Yang, Xinyu Wang, Yanru Lai, Yating Tang, Haoxiang Huang, Sifei Chen, Yanmei Chen

**Affiliations:** 1 The Second School of Clinical Medicine, Zhujiang Hospital, Southern Medical University, Guangzhou, China; 2 The School of Stomatology, Southern Medical University, Guangzhou, Guangdong, China; 3 The First School of Clinical Medicine, Southern Medical University, Guangzhou, Guangdong, China; 4 Department of Etiology and Carcinogenesis, National Cancer Center/National Clinical Research Center for Cancer/Cancer Hospital, Chinese Academy of Medical Sciences and Peking Union Medical College, Beijing, China; 5 Department of Cardiology, Guangdong Provincial Key Laboratory of Cardiac Function and Microcirculation, Nanfang Hospital, Southern Medical University, Guangzhou, China; 6 State Key Laboratory of Cardiovascular Disease, Fuwai Hospital, National Center for Cardiovascular Diseases, Chinese Academy of Medical Sciences and Peking Union Medical College, Beijing, China

**Keywords:** artificial intelligence, cardiovascular diseases, machine learning, multi-omics, precision medicine

## Abstract

Cardiovascular disease remains a major global health burden. Owing to its complex pathogenesis and marked clinical heterogeneity, conventional one-size-fits-all strategies often yield limited benefit for a substantial proportion of patients. Precision medicine advocates individualized management based on patients' clinical and molecular characteristics to improve outcomes. In this context, multi-omics and machine learning provide critical technical support for precision medicine: multi-omics can capture the full spectrum of cardiovascular disease from molecular alterations to phenotypic manifestations, while machine learning is well-suited to modeling complex associations between high-dimensional, nonlinear omics data and clinical outcomes. Their integration has therefore opened new avenues for the precise management of cardiovascular disease. This review systematically summarizes recent advances in applying machine learning and multi-omics to precision cardiovascular medicine, with a focus on three core domains: diagnosis, risk prediction, and treatment response prediction. In diagnosis, these approaches can assist with definitive diagnosis, early detection, differential diagnosis, and severity assessment, thereby enabling more noninvasive, objective, rapid, and precise evaluation of cardiovascular disease. In risk prediction, they support a comprehensive framework spanning primary prevention, secondary prevention, short-term risk stratification, and screening of high-risk populations, allowing risk management across the full disease course and across diverse patient groups. In addition, they enable individualized prediction of the benefits and risks of pharmacological and surgical treatments, thereby informing therapeutic decision-making. To facilitate clinical translation, several challenges remain particularly important, including data quality control, continuous model validation, improved transparency, clarification of responsibility and accountability, and supportive policies regarding implementation and cost coverage.

## 1 Introduction

Cardiovascular diseases (CVDs) remain the leading cause of death worldwide, accounting for approximately one-third of all deaths annually (1). Their pathogenesis is complex and involves the interplay of genetic, metabolic, inflammatory, and environmental factors. As a result, substantial interindividual heterogeneity exists in both clinical phenotypes and the underlying molecular mechanisms. Conventional diagnostic and therapeutic strategies often rely on overly generalized management paradigms that fail to capture this heterogeneity, leaving many patients without optimal clinical benefit (2). Accordingly, contemporary CVD management faces a central challenge: delivering more precise diagnosis, risk stratification, and individualized treatment for complex cardiovascular conditions (3).

Precision medicine seeks to integrate multidimensional information, including an individual's clinical, genetic, molecular, and environmental characteristics, to enable accurate disease characterization and risk stratification, facilitate tailored management, improve clinical outcomes, and reduce inefficient use of healthcare resources (4, 5). Its implementation depends on a comprehensive understanding of disease biology and the development of robust predictive models. The rapid evolution of multi-omics technologies and machine learning (ML) has provided an important technological foundation for advancing precision medicine in CVDs (6). Multi-omics approaches generate the molecular data required to characterize the biological and phenotypic complexity of CVDs at multiple levels (7). ML, as a powerful analytical framework, can uncover clinically relevant associations between high-dimensional omics data and patient outcomes. In addition, deep learning (DL) addresses several limitations of conventional machine-learning methods and enables more comprehensive analyses of intricate, unstructured, and high-dimensional data, including radiomics and single-cell omics (8, 9).

Against this background, this review systematically summarizes recent advances in applying multi-omics and ML to precision medicine for CVDs, focusing on three major domains. First, we examine CVD diagnosis, including improvements in diagnostic accuracy, early detection, differential diagnosis, and disease-severity assessment, and discuss how these approaches enhance disease identification. Second, we review risk assessment, encompassing primary and secondary prevention, short-term risk prediction, and risk stratification among high-risk populations, with particular emphasis on their implications for individualized clinical management. Third, we discuss predicting treatment response, emphasizing the potential of these approaches to forecast responses to pharmacological and procedural interventions, thereby informing the selection of optimal therapeutic strategies. Finally, we analyze the current limitations of the field and outline future opportunities for clinical translation.

## 2 Multi-Omics and machine learning overview

### 2.1 Multi-omics technology and multi-omics integration strategies

Multi-omics technologies encompass high-throughput molecular data generated by genomics, epigenomics, transcriptomics, proteomics, metabolomics, single-cell omics, and spatial omics, among others. These technologies can systematically elucidate the molecular mechanisms underlying the onset and progression of CVDs at the levels of genetic background, transcriptional regulation, protein function, metabolic status, cellular heterogeneity, and tissue heterogeneity. At the same time, radiomics, through the analysis of medical images, provides a macroscopic view of organ lesions and disease burden, effectively complementing molecular omics (10). Multi-omics can map a comprehensive panorama from the molecular to the phenotypic level, supplementing traditional clinical characteristics such as age, sex, and laboratory parameters, and enabling a more comprehensive and in-depth exploration of heterogeneity among individuals.

Currently, most models in this field remain based on a single omics approach, which may be limited by data collection costs and constraints of algorithmic analysis. However, true multi-omics integration research involves using computational methods to discover potential associations or complementary information across different omics disciplines. Although still in the early stages of development, this approach holds significant potential for elucidating the complex mechanisms of CVDs and advancing precision medicine. Given that existing evidence primarily stems from the application of ML based on single-omics data, this paper systematically summarizes the application of ML across various omics datasets and, based on this foundation, further explores strategies and future directions for multi-omics integration.

Based on different data fusion methods, multi-omics integration strategies are categorized into three models: early fusion, mid-stage fusion, and late-stage fusion. Early fusion is the most direct method of data integration, in which data matrices that have undergone preprocessing (e.g., standardization) are simply concatenated to form a unified high-dimensional matrix, followed by direct modeling and analysis. Mid-stage fusion, in contrast, uses feature representation learning to map different omics data into a common latent space, enabling dimensionality reduction while capturing the nonlinear interactions among different omics. Late-stage fusion, meanwhile, functions as a model ensemble for decision-making; it involves building independent models for each omics dataset and then obtaining the final prediction results through decision fusion or weighted prediction methods (11).

Each of these three integration approaches has its own advantages and limitations regarding data heterogeneity, model interpretability, scalability, and clinical translation; therefore, an appropriate fusion strategy must be selected based on specific requirements. Relatively speaking, the direct concatenation method of early fusion, while simple in its processing, lacks an explicit cross-omics learning mechanism. It has limited ability to capture complex omics interactions and is sensitive to scale differences across omics. As the number of omics dimensions increases, its complexity and risk of overfitting rise, leading to reduced generalizability and stability in practical applications. In contrast, mid-stage fusion is better suited for handling heterogeneous data from different platforms and for capturing associations between omics, but its complex computational strategies and architectural models limit interpretability and increase computational resource requirements, making it more suitable for exploratory research on cross-omics collaboration. Late-stage fusion, on the other hand, offers strong interpretability, high fault tolerance (results can still be produced even when a single modality is missing), and high computational scalability, which better align with the realities of incomplete data in real-world settings. However, since models for each omics modality are trained independently, late-stage fusion may not fully capture interactions between omics modalities (11–13).

### 2.2 Machine learning algorithms and applications

Omics data are characterized by high dimensionality, substantial noise, nonlinearity, and complex internal correlations; traditional statistical methods often fail to uncover key biological patterns. As a key branch and implementation method of artificial intelligence (AI), ML excels at automatically learning rules and patterns from large, complex data and generalizing them to new datasets. By leveraging this learning approach, models can establish subtle and intricate associations between omics data and outcome variables to perform probabilistic estimates for specific clinical scenarios (14).

Based on how training data are labeled, ML can be broadly categorized into supervised and unsupervised learning. Supervised learning primarily uses input features (e.g., omics data) and corresponding label information (outcomes, e.g., diagnostic results, mortality status) to identify optimal model parameters and construct models that accurately predict outcomes; it is therefore well suited for constructing diagnostic and predictive models. Problems can be categorized into classification and regression based on the label information. In classification, the model aims to predict discrete category labels for the input data, whereas in regression, the model predicts continuous values by fitting a regression function. The models applicable to these two types differ; regression often employs linear regression and regression trees. In contrast, unsupervised learning uses unlabeled data to explore hidden structures and patterns within the data, encompassing clustering and dimensionality reduction. Clustering divides samples into groups with similar characteristics, while dimensionality reduction focuses on simplifying and visualizing high-dimensional data. Consequently, unsupervised algorithms can analyze the heterogeneity of CVDs, reveal distributional structures within the data, uncover potential subtypes, and enable more accurate prognostic assessments and treatment planning (14).

Based on algorithm type, ML can be divided into traditional machine learning and deep learning. Traditional algorithms encompass classic methods such as support vector machines and decision trees, which are widely applied in cardiovascular omics modeling. Among these, ensemble models based on decision trees—including Random Forest (which reduces overfitting risk by aggregating multiple trees), Extreme Gradient Boosting (XGBoost), and Categorical Boosting (CatBoost) (which progressively fits residuals to improve predictive accuracy)—have demonstrated good accuracy and stability in numerous studies (15). Regularized linear models such as Least Absolute Shrinkage and Selection Operator (LASSO) and elastic net play a crucial role in screening high-dimensional omics features and can be used for preliminary dimensionality reduction and biomarker identification. DL, by contrast, employs multi-layer neural networks to learn representations and nonlinear transformations, enabling autonomous feature extraction and supporting tasks such as classification and regression, and it offers significant advantages in handling unstructured data (e.g., imaging data) (8). The relatively opaque “black box” nature of DL can reduce interpretability and affect model generalization and clinical translation; however, the use of attention mechanisms (such as Transformers) has enhanced its image analysis capabilities in recent years.

**Figure 1:**
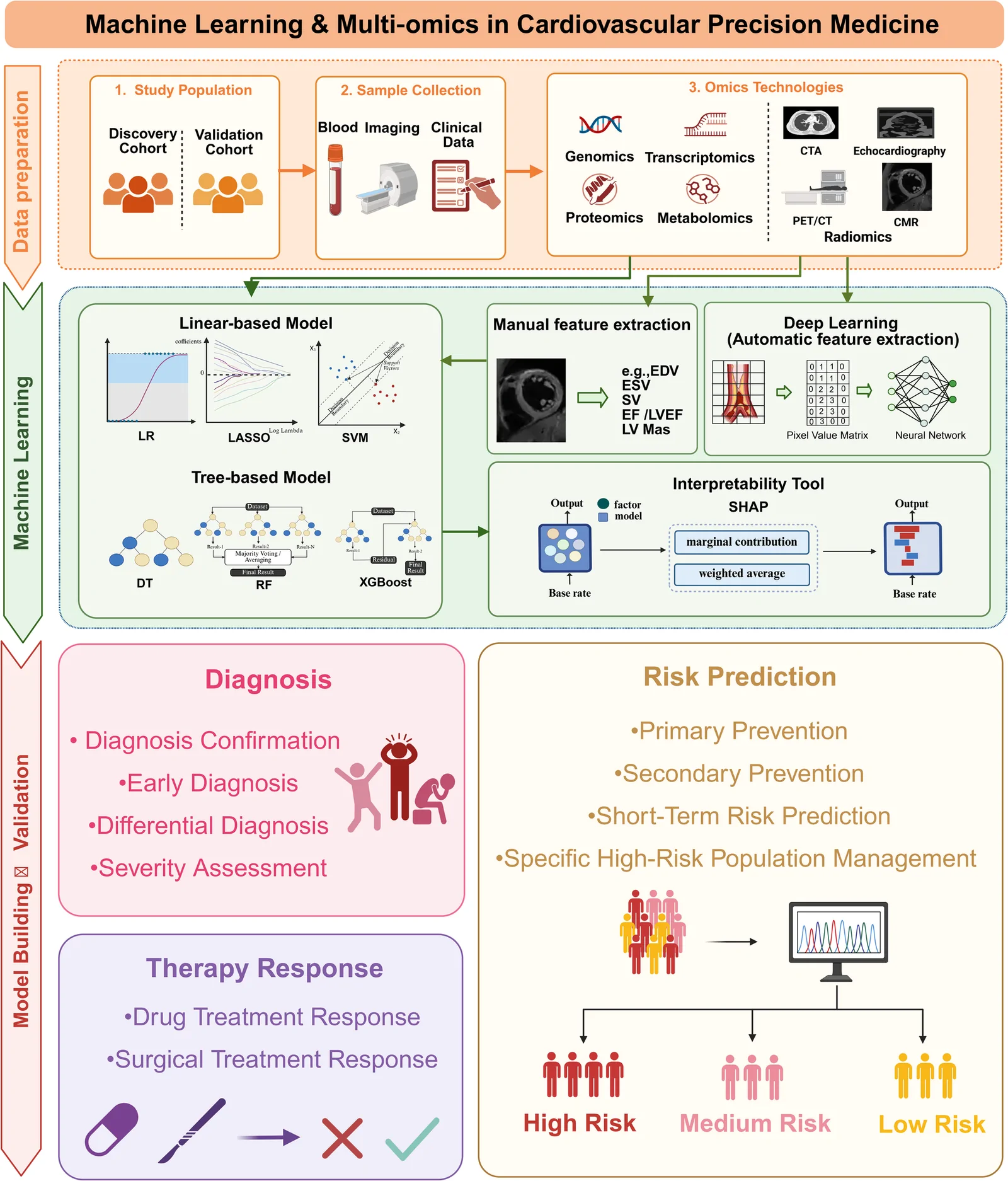
Framework of precision cardiovascular medicine driven by multi-omics and ML. This figure systematically illustrates the workflow and applications of multi-omics and ML in precision cardiovascular medicine research. **(1) Data preparation**. Blood samples, medical imaging data, and clinical information are collected via well-designed discovery and validation cohorts. Multi-omics technologies, including genomics, transcriptomics, proteomics, and metabolomics, are employed, and raw medical imaging data are processed according to standardized procedures to construct analytical datasets. **(2) Machine learning.** Appropriate machine learning algorithms are selected for subsequent analysis. Given the feature characteristics and dimensionality of omics data, conventional ML approaches (e.g., linear models such as LR and LASSO, and tree-based models such as RF and XGBoost) and DL frameworks are used to construct models. *Post-hoc* interpretability tools, such as SHAP, are further integrated to enhance model clarity and transparency. **(3) Model building & validation.** Established ML models can be implemented in the clinical practices of CVDs, comprising diagnosis assistance (diagnosis confirmation, early diagnosis, differential diagnosis, and disease severity assessment), risk prediction (primary prevention, secondary prevention, short-term risk prediction, and special high-risk population management), as well as therapy response prediction (evaluation of drug and surgical treatment responses). Collectively, multi-omics and ML serve as critical technical pillars for advancing cardiovascular precision medicine. CTA, computed tomography angiography; PET/CT, positron emission tomography/computed tomography; CMR, cardiac magnetic resonance; LR, logistic regression; LASSO, least absolute shrinkage and selection operator; SVM, support vector machine; DT, decision tree; RF, random forest; XGBoost, eXtreme gradient boosting; DL, deep learning; SHAP, SHapley additive explanations; CVD, cardiovascular disease; ML, machine learning; EDV, end-diastolic volume; ESV, end-systolic volume; SV, stroke volume; EF, ejection fraction; LVEF, left ventricular ejection fraction; LV Mass, left ventricular mass.

The multi-omics and machine-learning-driven framework for precision cardiovascular medicine is summarized in Figure 1.

## 3 Diagnosis

### 3.1 Disease confirmation

In the clinical diagnosis of CVDs, definitive diagnosis for certain conditions still relies heavily on invasive procedures. These methods carry risks of injury, incur substantial costs, and hinder dynamic monitoring and long-term follow-up. Although relatively mature noninvasive assessment methods exist, their accuracy is often limited by various factors; for example, plaque calcification may lead operators to overestimate the degree of coronary stenosis (16).

Against this backdrop, ML can analyze various imaging modalities to objectively quantify key indicators in noninvasive examinations, thereby effectively reducing the subjective influence arising from operator-dependent semi-quantitative interpretations or differences in observation planes. Taking the diagnosis of coronary heart disease as an example, invasive coronary angiography (ICA) remains the clinical gold standard; while CT coronary angiography (CCTA) offers a noninvasive alternative to assess anatomical stenosis of the coronary arteries, the consistency of manually estimated stenosis severity is poor, particularly in the moderate stenosis range of 30% to 70% (17). DL can directly analyze CCTA images to improve diagnostic stability and accuracy while reducing reading time. Lin et al. developed an automated coronary stenosis grading system using CCTA images from 921 patients recruited across multiple international centers, with expert consensus serving as the reference standard. The results showed that within a clinically acceptable margin of error (i.e., a difference of no more than one CAD-RADS grade), the model achieved 100% agreement with expert consensus in assessing the degree of vascular stenosis, demonstrating strong stability, while its agreement with ICA findings reached 97%, indicating good diagnostic accuracy; in terms of efficiency, the system significantly reduced the time required for plaque analysis per patient from the average 25.66 min taken by experts to 5.65 s (18).

In contrast, because functional testing cannot directly provide anatomical evidence, its results remain subject to systematic errors. A large-scale survey involving 660,000 patients with coronary heart disease showed that among patients with abnormalities detected by functional tests such as positron emission tomography/computed tomography (PET/CT), 55%–56% were ultimately found to have no stenosis upon ICA confirmation (19). ML, however, can predict anatomical lesions by learning deep features from functional images, thereby bridging the gap between functional and anatomical findings. To address this limitation, Betancur et al. were the first to develop a correlation model between raw PET/CT polar plots and stenosis results determined by ICA. They achieved effective discrimination of obstructive stenosis in 4,914 arteries across 1,018 patients, with diagnostic performance superior to that of traditional multiparametric imaging methods (AUC 0.80 vs. 0.78) (20). Subsequently, multiple studies have further optimized this approach, including improvements in image segmentation and data augmentation (21), CT-based PET/CT image correction (22), incorporation of clinical features (23), integration of supine and upright images (24), and multi-model comparisons (25). Conversely, by learning from anatomical changes, it is possible to predict functional indicators through correlation analysis. For example, one study correlated anatomical parameters—such as RVWT (right ventricular wall thickness) extracted from cardiovascular magnetic resonance (CMR)—with mean pulmonary artery pressure (mPAP) values obtained via right heart catheterization, thereby enabling the prediction of invasive functional indicators using noninvasive anatomical information. This model was built using the interpretable Sure Independence Screening and Sparsifying Operator (SISSO) algorithm and can output clear, symbolic formulas, facilitating clinical translation (26).

In these applications, DL algorithms [e.g., convolutional neural networks (CNNs) and long short-term memory networks (LSTMs)] serve as central analytical tools; however, their inherent “black-box” nature remains a major obstacle to clinical trust. Against this backdrop, interpretable methods such as “attention maps” can provide more objective and visual evidence to support clinical decision-making. For example, physicians can intuitively observe the specific areas the algorithm focuses on when assessing the degree of stenosis (e.g., plaque features or luminal margins), thereby enhancing their understanding of and trust in the model's judgments (27).

**Figure 2:**
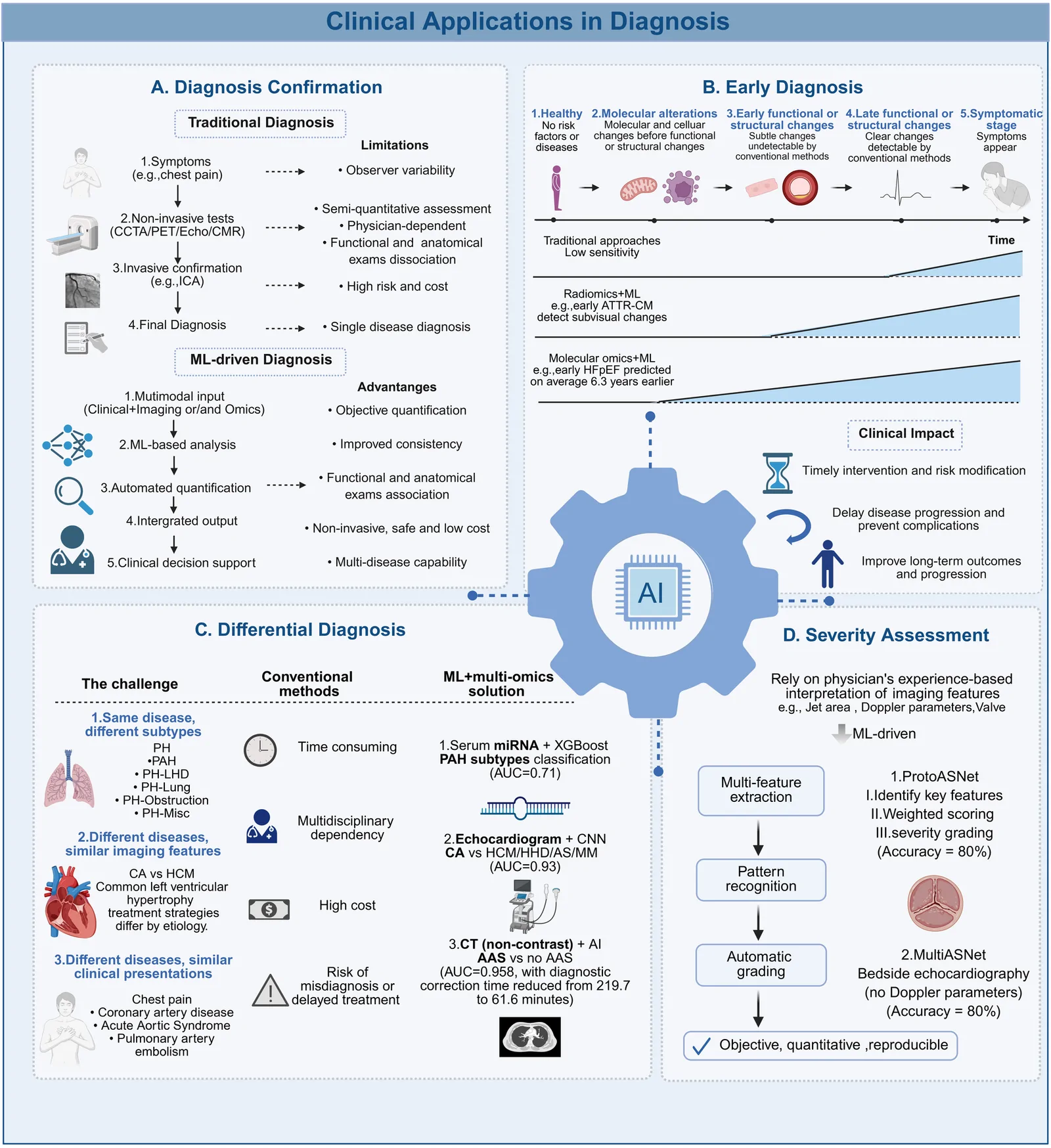
Clinical applications of multi-omics and ML in the diagnosis of CVDs. This figure illustrates the pivotal value of multi-omics and ML in CVD diagnosis in four aspects: confirmatory diagnosis, early diagnosis, differential diagnosis, and severity assessment. **(A)** Diagnosis Confirmation. Traditional diagnostic workflows for certain CVDs largely rely on invasive examinations, whereas non-invasive modalities are limited by semi-quantitative evaluation and the disconnection between functional and anatomical evidence. ML can effectively mitigate these limitations and facilitate comprehensive clinical management of cardiovascular comorbidities. **(B)** Early diagnosis. ML enables the detection of molecular perturbations at the subclinical stage via molecular omics profiling and captures subtle textural alterations in imaging data to identify minimal early structural and functional abnormalities. This strategy allows preclinical recognition of asymptomatic CVDs and provides a critical time window for early therapeutic intervention (29, 30). **(C)** Differential diagnosis. ML can distinguish different subtypes of the same disease, as well as distinct diseases with overlapping imaging phenotypes or clinical manifestations. With robust classification performance, it clarifies diagnostic decision-making, shortens the time to a definitive diagnosis, and streamlines conventional diagnostic workflows (31–33). **(D)** Severity assessment. By implementing multi-feature extraction and pattern recognition, ML enables automated, objective, and reproducible severity grading for valvular heart disease, CAPs, ventricular remodeling, and other pathological changes, thereby improving the consistency and reliability of quantitative evaluation (34, 35). CVDs, cardiovascular diseases; CCTA, coronary computed tomography angiography; PET, positron emission tomography; Echo, echocardiography; CMR, cardiac magnetic resonance; ICA, invasive coronary angiography; ATTR-CA, transthyretin cardiac amyloidosis; HFpEF, heart failure with preserved ejection fraction; PH, pulmonary hypertension; PAH, pulmonary arterial hypertension; PH-Lung, pulmonary hypertension due to lung disease; PH-LHD, pulmonary hypertension due to left heart disease; PH-Misc, pulmonary hypertension due to miscellaneous causes; miRNA, micro ribonucleic acid; CA, cardiac amyloidosis, CAP, coronary atherosclerotic plaque; HCM, hypertrophic cardiomyopathy; AAS, acute aortic syndrome; AI, artificial intelligence; ML, machine learning.

Furthermore, in clinical practice, patients often present with complex scenarios involving multiple coexisting conditions. For example, patients with hereditary cardiomyopathy frequently have concomitant valvular disease, while those with ventricular thrombus often exhibit myocardial ischemia and heart failure (HF). This disease complexity and the multifaceted clinical manifestations not only affect diagnostic and therapeutic quality across healthcare institutions but also place higher demands on the interpretive capabilities of algorithms. By optimizing omics-based and machine-learning approaches, these methods may provide robust support for comprehensive and high-quality clinical management. For instance, one study improved upon traditional multi-disease diagnostic models by using cardiac MRI images to train independent binary classification models for 39 CVDs—including hypertrophic cardiomyopathy, ventricular thrombus, and valvular heart disease—thereby enabling simultaneous diagnosis of multiple conditions. Notably, this model is based on the advanced Transformer architecture and was trained using a self-supervised contrastive learning strategy that combines imaging with text reports, enabling it to understand pathological anatomical changes and demonstrating excellent generalization performance in highly heterogeneous and complex clinical scenarios (28). At present, the exploration of ML and multi-omics in the diagnosis of cardiovascular comorbidities remains in its early stages. Future research could focus on specific clinical scenarios to develop diagnostic models tailored to particular combinations of comorbidities, thereby enabling more accurate identification of comorbidities and personalized treatment. Clinical applications of multi-omics and ML in CVD diagnosis are summarized in Figure 2.

### 3.2 Early diagnosis

The goal of early diagnosis of CVDs is to identify asymptomatic or subclinical patients with relevant risk factors, enabling timely intervention, delaying disease progression, and improving prognosis. This strategy is particularly important for conditions such as subclinical, occult myocardial injury, early asymptomatic cardiomyopathy, cardiac amyloidosis (CA), and pulmonary arterial hypertension (PAH), for which sensitive early assessment methods are currently lacking. However, current clinical practice largely relies on routine blood biomarkers, electrocardiograms, and echocardiograms for screening; these methods have low sensitivity and struggle to accurately identify patients in the early stages of the disease (36).

Before the emergence of clear structural or functional abnormalities in CVDs, cardiomyocytes, endothelial cells, and other cardiovascular cell types may already exhibit molecular dysregulation at the level of biological pathways, such as energy metabolism, inflammatory responses, and oxidative stress, as reflected in plasma molecular characteristics (37). ML models that integrate molecular omics data can detect subtle molecular changes in the early stages of disease onset and progression, enabling the identification of individuals with asymptomatic or subclinical CVDs even before abnormalities are detected through routine examinations. Versnjak et al. analyzed clinical phenotypic, metabolomic, and proteomic data from 401,917 individuals with high-risk factors. Using similarity network fusion (SNF) for multi-omics integration, they constructed the CatBoost model. Compared with cardiac MRI and N-terminal pro-B-type natriuretic peptide (NT-proBNP), this model can identify patients who will progress to HF with preserved ejection fraction an average of 6.3 years earlier in asymptomatic individuals (AUC = 0.931) and has been validated across 22 non-overlapping subsets. This model shifts the diagnostic window for HF from the symptomatic stage (Stage C) to the preclinical stage (Stage B), providing a time window for early intervention (38). Beyond single-disease applications, spectral classification of “molecular cargo” in machine-learning-driven extracellular vesicles also offers a promising pathway for universal early screening of multiple CVDs (29).

Similarly, traditional imaging typically relies on interpreting structural or functional abnormalities in the heart that are visible to the naked eye; however, by the time these abnormalities become apparent, the disease has often progressed to the middle or late stages of the subclinical phase. In contrast, ML can extract quantitative features from images that are difficult for the human eye to detect, capturing subtle changes associated with the early stages of the disease, thereby potentially shifting the diagnostic window earlier. Transthyretin-related cardiac amyloidosis (ATTR-CA) provides a representative example: its early manifestations often resemble those of left ventricular hypertrophic diseases such as HCM. Furthermore, the granular shine sign in the myocardial wall relies on manual interpretation, making it difficult to identify early, subtle infiltrations; consequently, patients are often diagnosed at a later stage. Although Mori et al. primarily built their model using pathologically confirmed data from patients with mid-to-late-stage ATTR-CA, they successfully used echocardiographic texture features to quantify subclinical granular changes that are difficult for the human eye to detect, demonstrating excellent diagnostic performance (AUC = 0.97–0.99) in a multicenter dataset. Although this technology still requires further validation in prospective early-stage cohorts, the results have preliminarily indicated that image-based analysis methods hold promise for shifting the diagnostic window for ATTR-CA to earlier stages of the disease (30).

Currently, the implementation of large-scale screening and long-term follow-up in asymptomatic high-risk populations remains constrained by practical factors, including high testing costs, complex implementation processes, and significant barriers to widespread adoption. At the same time, while using follow-up outcomes as the primary evaluation metric can indirectly validate clinical value, it primarily reflects long-term risk prediction and struggles to characterize the disease state during the subclinical stage directly. In the future, if long-term follow-up data can be integrated with surrogate markers that accurately characterize the subclinical state, the scope for exploration and translational potential of multi-omics combined with ML in the early identification and precision intervention of CVDs remains vast.

### 3.3 Differential diagnosis

In cardiovascular medicine, certain diseases exhibit overlapping clinical presentations and similar imaging features, yet their pathophysiological mechanisms and treatment strategies differ significantly. Examples include CA vs. HCM, secondary vs. primary hypertension (PHT), different subtypes of PAH, and acute chest pain or syncope caused by various etiologies. Traditional differential diagnosis often relies on complex, multi-step clinical workflows and requires multidisciplinary collaboration, which is costly and can lead to treatment delays or difficulties in achieving precise treatment due to low patient compliance. In contrast, ML can leverage high-dimensional data spaces constructed from multi-omics data to identify optimal classification hyperplanes among disease groups with similar phenotypes, accurately capturing subtle differences between them, thereby providing technical support for simplifying the differential diagnosis pathway.

First, the integration of multi-omics and ML provides a more efficient technical approach for distinguishing different subtypes of the same disease. Taking PAH as an example, traditional subtyping requires a combination of multiple tests, such as CTPA, pulmonary function tests, and ultrasound, and relies on multidisciplinary collaboration involving pulmonology and rheumatology departments, making the process complex and time-consuming. Errington et al. studied 1,150 patients with different subtypes of pulmonary hypertension who had been diagnosed using standard criteria, along with 334 healthy controls. Based on serum microRNA omics data, they used XGBoost for feature selection and constructed a generalized linear model, successfully achieving the diagnosis of pulmonary hypertension and the effective differentiation of its various subtypes. The discriminatory performance of this model was significantly superior to that of the traditional biomarker NT-proBNP (AUC = 0.71 vs. 0.49) (31). Similarly, distinguishing between primary and secondary hypertension often requires multiple tests, such as hormone assays and imaging studies. In a study integrating multi-omics features from plasma and urine, various supervised learning algorithms were applied to model and compare 487 patients with clearly classified hypertension. In the binary classification of endocrine hypertension (EHT) vs. PHT, logistic regression performed best (AUC = 0.96), whereas in the four-class classification of pheochromocytoma/functional paraganglioma, primary aldosteronism, Cushing syndrome, and PHT, the random forest model performed best (AUC = 0.95) (39). In CA, treatment strategies differ between light-chain and transthyretin subtypes, but differential diagnosis relies on myocardial biopsy. Hong et al. included pathologically classified patients and constructed a random forest model using 38 imaging features derived from the fusion of ^11^C-PiB PET and ^99^mTc-DPD, achieving rapid differentiation of CA subtypes (AUC = 0.94) (40).

Certain CVDs share highly similar imaging features, making them difficult to distinguish accurately on routine examinations and leading to unnecessary invasive procedures or delays in treatment. For example, conditions such as CA and HCM that cause left ventricular hypertrophy often show overlapping echocardiographic findings. One study analyzed transthoracic echocardiograms from 1,349 patients with standard-diagnosed CA, using cases of hypertensive cardiomyopathy, aortic stenosis (AS), HCM, and multiple myeloma as controls. The model trained using a CNN demonstrated good diagnostic performance for CA in external validation (AUC = 0.93) (32). Another study focused exclusively on differentiating CA from HCM and achieved highly accurate discrimination between the two using CMR data (AUC = 0.99) (41).

Clinical symptoms such as chest pain and syncope often stem from complex and diverse etiologies. An efficient differential diagnosis process can rapidly narrow the differential diagnosis and facilitate timely intervention. Take acute chest pain as an example: its causes include coronary artery disease (CAD), particularly acute coronary syndromes such as ST-elevation myocardial infarction (STEMI) and non-ST-elevation myocardial infarction (NSTEMI), as well as aortic dissection and pulmonary embolism. While the clinical presentations are highly similar, the treatment strategies differ significantly. Routine emergency department management typically involves sequentially ruling out these conditions using electrocardiograms, troponin and D-dimer tests, and imaging studies—a time-consuming process that carries a risk of misdiagnosis and potential contrast-related complications. Against this backdrop, a multicenter study developed the AI-based early warning system iAorta. By modeling non-contrast CT scans from patients with guideline-defined acute aortic syndrome (AAS) and non-AAS patients, the system can identify AAS patients without the need for contrast agents (AUC = 0.958). In a prospective evaluation across eight hospitals, the system significantly reduced the time to correct initial misdiagnoses from 219.7 min to 61.6 min (33). Furthermore, Fan et al. used plasma metabolomic data to develop a logistic regression model to distinguish nonobstructive coronary atherosclerosis, normal coronary artery, stable angina, unstable angina, and acute myocardial infarction (AMI) in patients with acute chest pain. The model performed well, with the binary classification model for distinguishing unstable angina from AMI yielding the best results (AUC = 0.992) (42).

### 3.4 Severity assessment

Assessment of disease severity in certain cardiovascular conditions relies heavily on physicians' clinical experience and subjective judgment, which may lead to disparities in assessment quality and consistency between specialized cardiac centers and primary care facilities. Therefore, the introduction of multi-omics technologies—such as radiomics—and ML-driven automated quantitative assessment tools can effectively address the shortage of specialized resources in primary care, providing a more objective, stable, and reproducible basis for grading the severity of CVDs, as well as for determining the optimal timing of treatment and developing treatment plans.

As an example, in the case of valvular disease, traditional assessments rely on physicians' expert interpretation of imaging features such as regurgitant jet area, spectral shape and velocity, valve orifice shape and area, chamber dimensions, and ventricular wall thickness and motion; these methods are susceptible to variations in image quality and subjective interpretation. In contrast, ProtoASNet uses typical lesions annotated by multiple physicians as a reference to identify key features such as calcification and restricted valve motion. It then calculates a weighted sum of these key features to provide a reliable assessment of aortic stenosis severity (accuracy = 80%) (34). MultiASNet, by contrast, is designed for resource-limited settings and enables the detection and grading of AS using bedside ultrasound without Doppler parameters (accuracy = 80%) (35). Additionally, machine-learning analysis of CT images can noninvasively quantify changes in patients' hemodynamic parameters, thereby indirectly assessing the severity of aortic valve disease (43). In the field of mitral valve disease, Vrudhula et al. developed a DL model based on color Doppler echocardiography, using a multi-expert consensus as the standard, which effectively identifies moderate-to-severe (AUC = 0.951) and severe (AUC = 0.969) mitral regurgitation (44). Similar methods have also been used to achieve automated severity grading of mitral valve prolapse (45).

The automated assessment models described above can provide a more objective basis for the diagnosis and treatment of valvular disease, but their clinical implementation still requires prospective validation and expert-consensus evaluation. In addition to valvular disease, this technical approach applies to the objective, quantitative analysis of key indicators, including coronary atherosclerotic plaques, ventricular volume, and ejection fraction. Given that such assessment results are frequently used for risk stratification in CVDs, please refer to the relevant sections for further details. Representative studies using ML and multi-omics for CVD diagnosis are summarized in Table 1.

**Table 1:** Summary of representative studies using ML and multi-omics for CVD diagnosis.

Task	Condition	Omics/ Modality	Input	ML Algorithm(s)	Main outcome	Sample size	Validation type	Best performance	Risk of bias^a^	Applicability concern^a^	Reference
Disease Confirmation	CAD	Radiomics	CCTA	ConvLSTM	CAD-RADS grades	Discovery:921 Ext Val:275	Ext Val	κ = 0.78	Unclear risk	Low concern	(18)
Disease Confirmation	PH	Radiomics	CMR (10 features)	SISSO	mPAP prediction	Discovery:120	Int Val	AUC = 0.987	Unclear risk	Low concern	(26)
Disease Confirmation	Multiple CVDs (39 diseases)	Radiomics	CMR cine sequences & text reports	Transformer	Multi-disease classification	Discovery: 45,623 Ext Val: 9,719	Ext Val	AUC = 0.65–0.97	Unclear risk	Low concern	(28)
Early Diagnosis	HFpEF	Metabolomics, Proteomics	Clinical phenotypes, metabolomics, proteomics	SNF + CatBoost	HFpEF detection	Discovery: 401,917 Validation: 100,446	Ext Val	AUC = 0.931	Unclear risk	Unclear concern	(38)
Early Diagnosis	ATTR-CA	Radiomics	Echocardiographic texture features (94 features)	Logistic Regression	ATTR-CA detection	Discovery: 453 Ext Val: 64	Ext Val	AUC = 0.97–0.99	Low risk	Low concern	(30)
Differential Diagnosis	CA	Radiomics	^11^C-PiB PET, ^99^mTc-DPD (38 features)	Random Forest	CA subtype classification (AL vs. ATTR)	Discovery:27	Int Val	AUC = 0.94	High risk	Unclear concern	(40)
Differential Diagnosis	CA	Radiomics	TTE	CNN	CA vs AS/HFpEF/HCM/HHD/MM/MGUS	Discovery:2,612 Ext Val: 2,719	Ext Val	AUC = 0.93	Low risk	Low concern	(32)
Differential Diagnosis	AAS	Radiomics	Non-contrast CT scans	U-Net+Multi-task CNN	AAS detection	Discovery:2,287 Ext Val:17,163	Ext Val	AUC = 0.958	Unclear risk	Low concern	(33)
Severity Assessment	AS	Radiomics	Echocardiography	Prototype-based models	AS severity	Discovery: 2,572 Ext Val:599	Ext Val	Accuracy = 80.8%	High risk	Unclear concern	(34)
Severity Assessment	MR	Radiomics	Color Doppler echocardiograms	CNN	MR severity grading	Discovery: 38,461	Ext Val	AUC = 0.951 for ≥moderate; AUC = 0.969 for severe	Unclear risk	Low concern	(44)

ConvLSTM, convolutional long short-term memory; CAD, coronary artery disease; CAD-RADS, coronary artery disease reporting and data system; Ext Val, external validation; Int Val, internal validation; *κ*(kappa), Cohen's kappa; PH, pulmonary hypertension; CCTA, coronary computed tomography angiography; CMR, cardiac magnetic resonance; SISSO, sure independence screening and sparsifying operator; mPAP, mean pulmonary artery pressure; CVD, cardiovascular disease; SNF, similarity network fusion; CatBoost, categorical boosting; ATTR-CA, transthyretin cardiac amyloidosis; CA, cardiac amyloidosis; AL, light chain amyloidosis; TTE, transthoracic echocardiography; CNN, convolutional neural network; AS, aortic stenosis; HHD, hypertensive heart disease; HCM, hypertrophic cardiomyopathy; MM, multiple myeloma; MGUS, monoclonal gammopathy of undetermined significance; CT, computed tomography; AAS, acute aortic syndrome; U-Net, U-shaped convolutional neural network; MR, mitral regurgitation; ML, machine learning; AUC, area under the curve.

a Risk of bias and applicability concerns were assessed using author-adapted criteria informed by the PROBAST framework. Detailed assessment criteria are provided in Supplementary Tables S1 and S2.

## 4 Risk prediction

### 4.1 Primary prevention risk prediction

Primary prevention targets individuals who have not yet been diagnosed with CVDs and have not experienced clinical events. The primary objective is to identify individuals at high risk of developing atherosclerotic cardiovascular disease (ASCVD), HF, or other cardiovascular outcomes, while minimizing harm and ensuring wide applicability. Conventional risk scores such as Systematic COronary Risk Evaluation 2 (SCORE2) and Framingham have been demonstrated to predict the 10-year risk of ASCVD or cardiovascular events. Despite their ease of application, these models primarily rely on a limited set of clinical variables, such as age, blood pressure, and total cholesterol, and thus fail to accurately reflect complex biological processes, including inflammation and metabolism. Consequently, their predictive accuracy is limited. In contrast, multi-omics and ML algorithms can identify combinations of features that are more closely associated with future events by integrating features from multiple data modalities, including proteomic, metabolomic, microbiome, genetic, and incidental imaging data. This enhanced ability to predict long-term risk is a significant advancement in the field (46–50).

Of these, plasma proteomics is one of the areas with the most established evidence to date. To address how to identify hidden high-risk individuals missed by traditional scoring systems in event-free populations, Helgason et al. measured 4,963 plasma proteins in 13,540 individuals with no major ASCVD events at baseline and constructed a protein-based risk score using LASSO-penalized Cox regression. The findings indicated that this score showed a substantial correlation with the risk of a first ASCVD event. In a primary prevention test set, the model incorporating proteomic information exhibited superior predictive performance compared with the purely clinical model, with C-statistics of 0.747 and 0.736, respectively (51). Similarly, a study integrating seven proteins—including growth differentiation factor 15, identified via ML—with clinical variables from SCORE2 demonstrated superior performance to traditional clinical models across various subgroups in predicting myocardial infarction (MI) risk. Notably, the model showed improved predictive capacity in traditionally low-risk populations, such as individuals with high-density lipoprotein ≥40 mg/dL [net reclassification index (NRI) = 0.087]. Another study used a similar approach to improve the prediction of major vascular events, including aortic aneurysms and aortic dissections. The extant literature demonstrates that large-scale proteomics can provide incremental information for traditional risk assessment by supplementing molecular-level dimensions. Moreover, this approach facilitates the macro-level identification of high-risk populations for primary prevention of CVDs. Additionally, it can be refined to identify specific events that require prevention and the underlying biological pathways (52, 53).

**Figure 3:**
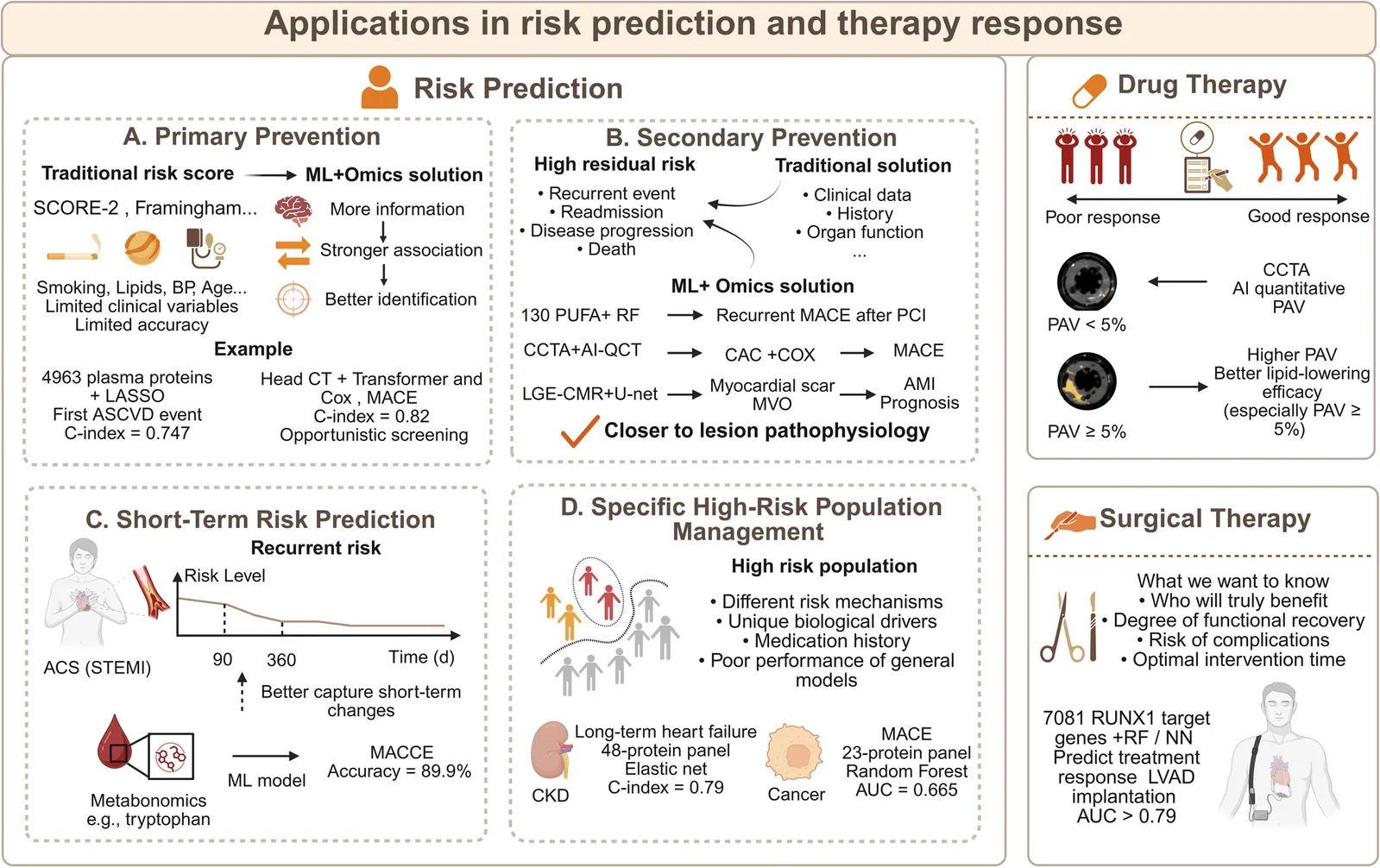
Applications of multi-omics and ML in CVD risk prediction and therapy response assessment. This figure illustrates the clinical translation value of multi-omics and ML from two angles: risk prediction and therapeutic response assessment. Left panel: Risk prediction **(A)** Primary prevention. This analytical framework provides incremental prognostic information beyond conventional clinical risk scores such as SCORE2, enabling reliable identification of high-risk individuals. It also offers a feasible methodological approach to opportunistic screening for CVDs (51, 54). **(B)** Secondary prevention. Secondary prevention aims to assess residual risk among patients with established CVD. Multi-omics, integrated with ML, enables characterization of pathophysiological processes closely linked to disease progression, thereby providing more comprehensive evidence for refined risk stratification and clinical management (55–57). **(C)** Short-term risk prediction. ML models based on metabolomic signatures capture acute pathophysiological activity and provide early warning of recurrent adverse events. This is particularly critical for high short-term-risk conditions such as ACS (58). **(D)** Specific high-risk population management. In populations with CKD and cancer, their underlying disease and medication exposure act as confounders to the models. They may compromise the predictive performance and inhibit accurate cardiovascular risk assessment. Tailored modeling based on multi-omics and ML can address the limitations of universal models and capture key disease-specific biological pathways in these vulnerable groups (59, 60). Right panel: Therapy response **(1)** Drug therapy. This approach enables individualized prediction of treatment benefits. For instance, quantitative assessment of percent atheroma volume enables precise evaluation of the net clinical benefit of lipid-lowering therapy (61). **(2)** Surgical therapy. Multi-omics and ML support a comprehensive evaluation of potential beneficiaries, the magnitude of functional recovery, the risk of complications, and optimal intervention timing. Representative applications include screening candidates with favorable functional recovery after LVAD implantation, providing evidence-based reference for personalized clinical decision-making (62) LASSO, least absolute shrinkage and selection operator; CVD, cardiovascular disease; ASCVD, atherosclerotic cardiovascular disease; CT, computed tomography; MACE, major adverse cardiovascular events; PUFA, polyunsaturated fatty acid; PCI, percutaneous coronary intervention; CCTA, coronary computed tomography angiography; CAC, coronary artery calcium; LGE-CMR, late gadolinium enhanced cardiac magnetic resonance; MVO, microvascular obstruction; AMI, acute myocardial infarction; ACS, acute coronary syndrome; STEMI, ST-segment elevation myocardial infarction; ML, machine learning; MACCE, major adverse cardiovascular and cerebrovascular events; CKD, chronic kidney disease; RF, random forest; NN, neural network; LVAD, left ventricular assist device.

Furthermore, the integration of radiomics and ML offers new insights for the primary prevention of CVDs. For instance, cranial CT, a widely utilized imaging modality in neurology and emergency medicine, offers a promising avenue for screening for CVDs. Zhang et al. utilized a Transformer model to analyze head CT scans and predict CVD events. When evaluated against the American Heart Association (AHA)-published PREVENT risk score, the model demonstrated superior classification performance (C-index = 0.82 vs. 0.75). Their innovative use of non-cardiac imaging data for assessment not only broadens the clinical perspective but also offers a feasible solution for large-scale real-world screening at a low external cost (54). Applications of multi-omics and ML in CVD risk prediction and therapeutic response assessment are summarized in Figure 3.

Beyond proteomics and radiomics, ML has also facilitated the use of gut microbiome features for primary cardiovascular prevention. In a multiethnic cohort of 3,860 participants without a history of CVDs, Warmbrunn et al. used an XGBoost model to predict the Framingham Risk Score from gut microbial features. The model achieved an overall root mean square error of 0.386 ± 0.006 and identified microbial networks involving Christensenellaceae, Methanobrevibacter, and Ruminococcaceae that were negatively associated with blood lipid levels and cardiovascular risk (63). These findings illustrate the potential of ML to derive clinically relevant risk signatures from high-dimensional microbiome data, identify metabolic subtypes, and construct biological aging indices. Nevertheless, model performance may vary across populations because of differences in genetic background, lifestyle, microbiome composition, and baseline risk. Multicenter prospective studies using incident cardiovascular events as clinical endpoints are therefore needed to establish generalizability and clinical utility.

### 4.2 Secondary prevention risk prediction

Secondary prevention targets individuals who have been diagnosed with CVD or have a history of cardiovascular events. The main objective of the study is to identify those at high risk of recurrence, disease progression, rehospitalization, or death during long-term follow-up. Compared with primary prevention, secondary prevention emphasizes residual risks and prognostic disparities throughout the disease course; therefore, it is necessary to integrate omics signals with prior medical history, treatment exposure, and organ damage status into the model. In the context of contemporary research, cardiovascular prognosis assessment predominantly employs hard endpoints, namely all-cause mortality, cardiovascular mortality, rehospitalization, and major adverse cardiovascular events (MACE), defined as cardiac death, nonfatal MI, and nonfatal stroke, as model labels (58, 64–66).

In this context, the value of multi-omics data lies primarily in supplementing the limitations of traditional clinical follow-up at the molecular level, thereby further revealing biological residual risk beyond conventional risk factors (67–69). To illustrate this, consider the case of STEMI. Conventional secondary prevention strategies primarily rely on medical history, left ventricular function, and well-established risk factors to estimate the risk of MACE after percutaneous coronary intervention (PCI). Nevertheless, these indicators possess limited predictive capacity for recurrent MACE (70). To address this issue, Du et al. established a predictive model for recurrent MACE following PCI using data from 645 STEMI patients. They found that traditional cardiovascular risk factors exhibited no significant differences between patients with and without recurrence. In contrast, a model constructed based on 130 plasma polyunsaturated fatty acid-derived metabolites effectively predicted recurrence risk. Notably, a panel comprising 14 key features selected by multivariate random forest analysis demonstrated excellent predictive performance, with an accuracy of 91.5% (55). Conventionally, the prognosis of infective endocarditis has been determined by the presence of specific blood biomarkers and clinical expertise. However, following the integration of plasma and vegetative proteomics to construct a prognostic model by He et al. (AUC = 0.83), this model outperformed models based on conventional blood biomarkers (CBMs) (AUC = 0.76). Furthermore, the combined model exhibited an augmented discriminatory ability (AUC = 0.87) (71). Together, these studies suggest that omics approaches can further enhance the ability to stratify residual biological risk in secondary prevention.

Complementing omics, the unique advantage of radiomics lies in its ability to more intuitively reflect the actual burden of disease and the extent of functional impairment at the organ level. When combined with ML, it can provide more objective and accurate measurements of prognostic indicators, thereby expanding the scope of medical imaging data utilization. To illustrate this point, consider the case of ATTR-CA. In this condition, left ventricular outflow tract velocity-time integral (LVOT-VTI) is a crucial functional indicator. Indeed, LVOT-VTI has been shown to serve as an alternative to invasive stroke volume measurements, facilitating dynamic disease monitoring. However, the measurement of these parameters is currently reliant on manual operations, which is a cumbersome process that often yields inconsistent results. Venneri et al. utilized ML to automatically and quantitatively extract LVOT-VTI from echocardiograms, thereby providing a more convenient, rapid, and standardized method for assessing disease progression and predicting outcomes in ATTR-CA (72). At the information dimension level, conventional imaging methods for assessing CAD place significant emphasis on the degree of stenosis. While this can intuitively reflect a patient's disease burden, it fails to adequately capture biological indicators or processes more closely associated with long-term prognosis, such as plaque composition, hemodynamic changes, and the extent of tissue damage. This results in an incomplete characterization. To address this shortcoming, Dahdal et al. used Food and Drug Administration-approved AI-quantitative coronary computed tomography analysis to perform automated quantitative analysis of coronary artery calcium (CAC) in CCTA images and correlated the scoring results with MACE. The findings demonstrated that, compared with stenosis grades CAD-RADS, CAC scoring substantially improved the prediction of MACE events (NRI = 0.47) (56). Furthermore, DL facilitates quantitative assessment of epicardial adipose tissue on CCTA, and while it correlates significantly with CAC scores, it offers independent prognostic value beyond CAC (73). These findings demonstrate the efficacy of this approach in facilitating the discovery and validation of new imaging biomarkers. Furthermore, the prognosis of patients with AMI is often influenced by multiple tissue damage factors, such as myocardial scarring and microvascular obstruction, while traditional biomarkers or left ventricular ejection fraction (LVEF) struggle to provide such a comprehensive set of information simultaneously. To address this limitation, Yang et al. developed the late gadolinium enhancement (LGE)-CMR net, a system based on the classic U-Shaped Convolutional Neural Network (U-Net) segmentation algorithm. This system can perform myocardial segmentation, scar assessment, and microvascular obstruction evaluation on LGE-CMR, thereby providing more objective and comprehensive long-term prognostic information for patients with AMI (57). The extant literature demonstrates that the true value of radiomics lies not merely in “examining images in greater detail” but in transforming imaging information, which traditionally relies on expert interpretation, into prognostic indicators that are closer to the essence of the pathology, quantifiable, reproducible, and integrable. This, in turn, enhances the stability and clinical applicability of risk stratification for secondary prevention.

In addition to supervised learning, unsupervised learning also offers important insights for long-term risk stratification in secondary prevention. Several studies have previously examined the use of unsupervised learning techniques for clustering proteomic or multimodal data in conditions such as HF and cardiomyopathy. These studies have demonstrated the potential to identify prognostic subtypes (74–79). The primary advantage of this approach is that it overcomes the limitations of traditional diagnostic labels, which can result in a homogeneous approach to patient management. This, in turn, allows the identification of outcome differences within the same disease that are driven by distinct mechanisms. In the context of CAD, analogous strategies have also demonstrated significant value: Guo et al. used unsupervised consensus clustering to categorize patients with poor coronary collateral circulation into two distinct phenotypes: Complement-Driven Vascular Remodeling and Immuno-Thrombotic Myocardial Dysfunction (AUC > 0.91). The latter phenotype exhibited a significantly higher incidence of MACE than the former (77). This finding indicates that subsequent secondary prevention initiatives should concentrate not only on identifying “high-risk patients” but also on ascertaining the specific high-risk mechanisms that precipitate adverse outcomes. This would provide a foundation for long-term follow-up and adjustments to the intensity of the intervention.

The paradigm of secondary prevention has shifted from merely identifying high-risk patients to more precisely delineating residual risk factors. This evolution is facilitated by integrating molecular characteristics, organ phenotypes, and disease subtypes, thereby providing a foundation for developing long-term follow-up strategies and adapting intervention intensity.

### 4.3 Risk stratification for acute events

Risk stratification for acute events emphasizes short-term prediction of mortality, reinfarction, worsening HF, stroke, or rehospitalization within approximately one year. In contrast to the assessment of long-term risk, the evaluation of short-term stratification places greater emphasis on dynamic biomarkers that reflect current pathological activity. Such biomarkers may include ischemia-reperfusion injury, inflammatory activation, coagulation abnormalities, microvascular obstruction, and scar burden.

In patients diagnosed with acute coronary syndrome (ACS), particularly those with acute myocardial infarction (STEMI/AMI), the challenge in short-term risk assessment lies in the fact that, while traditional clinical indicators can reflect the severity of the underlying condition, they often fail to capture in a timely and comprehensive manner the dynamic pathological information closely associated with recent deterioration. By comparison, metabolomic or proteomic signatures derived from plasma can reflect the dynamic molecular state during the acute phase. Furthermore, quantitative imaging can characterize the extent of organ damage and functional impairment. In the context of STEMI, Du et al. developed stroke and major adverse cardiac and cerebrovascular events risk prediction models based on tryptophan metabolites, achieving 89.9% predictive accuracy in 401 patients. This suggests that specific metabolic abnormalities can serve as early warning signals for short-term adverse outcomes in the acute phase (55, 58). For patients with AMI, Yang et al. adopted an organ imaging-phenotype approach, using a DL model based on LGE-CMR to automatically quantify myocardial scarring and microvascular obstruction, thereby enabling effective stratification of short-term adverse events (57). Regarding the risk of ischemia associated with ACS, Yu et al. further integrated quantitative coronary artery myocardial perfusion and CCTA phenotypes to construct a model, suggesting that subsequent risk is not determined solely by clinical severity but is also closely related to a comprehensive pattern of perfusion defects, lesion extent, and plaque micro-features (80). These studies complement short-term risk prediction in the acute phase from the perspectives of omics and organ imaging, respectively. This suggests that future risk stratification for acute events is more likely to rely on the combined modeling of dynamic molecular signals and quantitative imaging phenotypes.

### 4.4 Risk stratification in specific high-risk populations

In specific high-risk populations, such as patients with malignant tumors, chronic kidney disease, and autoimmune diseases like systemic lupus erythematosus, the mechanisms underlying CVD risk, the spectrum of events, and the biomarker profiles often deviate from those of the general population. This deviation is attributable to underlying diseases and the medications used to treat them. Consequently, models developed for the general population often lack sufficient transferability and calibration in these settings (59, 81). For these groups, the core objective of risk assessment is no longer limited to determining “who is more likely to develop the disease”; rather, it extends to elucidating why traditional models fail in specific pathological contexts, identifying additional biological processes driving risk, and optimizing monitoring and intervention strategies accordingly.

Recent studies have demonstrated that ML models based on lipid metabolomics, targeted proteomics, or large-scale proteomics exhibit superior risk-stratification capabilities compared with traditional clinical scoring systems in specific high-risk populations (81–85). For instance, among patients with chronic kidney disease (CKD), the incidence of HF is higher than that among individuals without CKD. In a particular study, an elastic net–Cox model incorporating 48 proteins outperformed a purely clinical model in predicting long-term HF (C-statistic = 0.79 vs. 0.70). Notably, the proteins identified by this model were predominantly enriched in the extracellular matrix remodeling pathway. This molecular signature has been demonstrated to reveal the causal link between CKD-associated hypertension and HF at the biological level (60). Among cancer survivors, proteomic risk assessment has similarly been shown to significantly improve predictive performance for major cardiovascular events. A study involving 4,225 cancer survivors demonstrated that a 23-protein panel identified by random forest analysis (AUC = 0.646–0.665) not only outperformed various traditional risk scores (AUC = 0.498–0.656) but also further improved the discriminatory power and risk reclassification capabilities of existing scores (59).

## 5 Prediction of treatment response

### 5.1 Prediction of response to pharmacological treatment

The major challenge in predicting response to pharmacological treatment is the significant variation in benefits and adverse effects among patients receiving the same standard therapy, while traditional clinical stratification often fails to identify those who will truly benefit before treatment. To address this need, existing studies have attempted to combine patient-specific omics features with ML to predict treatment responses in conditions such as HF, metabolic syndrome, PAH, and DCM (86–88). The common approach in these studies is to use current molecular status or disease burden to predict potential treatment benefits, thereby advancing “empirical drug therapy” to “individualized benefit assessment.”

In recent years, radiomics and AI-based quantitative analysis have also provided new insights for predicting responses to drug therapy. In CAD, the selection of conventional lipid-lowering therapy intensity is primarily based on clinical risk factors, low-density lipoprotein cholesterol (LDL-C) levels, and the degree of stenosis, making it difficult to distinguish further which patients are more likely to benefit from treatment. To address this limitation, Maaniitty et al. conducted an AI-guided quantitative CT analysis of 2,269 patients with suspected CAD. They performed quantitative analysis of CCTA images and used the atherosclerotic plaque volume fraction (PAV) to assess coronary plaque burden. The results showed that patients with higher PAV, particularly those with PAV > 5%, derived more significant prognostic benefits from lipid-lowering therapy (61). Consistent with this, the Determining the Value of Coronary CT Angiography (DECIDE) Registry further demonstrates that AI-based quantitative plaque analysis using CCTA not only allows for more precise quantification of atherosclerotic burden but also directly drives adjustments to preventive treatment strategies; the study shows that more than half of the patients underwent changes in management following receipt of AI analysis results, with the majority receiving new or intensified lipid-lowering therapy, accompanied by subsequent reductions in LDL-C levels (89). Together, these findings suggest that the value of AI-based quantitative plaque analysis lies not merely in “detecting more plaques” but in translating plaque-burden information into more targeted lipid-lowering treatment decisions. On the other hand, earlier but still representative omics studies have also shown that when pathological mechanisms are highly heterogeneous, treatment benefits are not entirely determined by traditional clinical stratification. For example, in PAH, a decision tree constructed from peripheral blood transcriptomic features correctly identified all 5 of 5 vasodilator-responsive PAH patients who demonstrated a long-term response to calcium channel blockers in an external validation cohort, suggesting that molecular features hold promise for predicting treatment response (90). These studies have advanced the prediction of treatment response from the perspectives of imaging lesion burden and molecular heterogeneity, suggesting that future efforts should focus on developing models to assess treatment benefit that are both interpretable and clinically applicable.

### 5.2 Predicting response to surgical treatment

Compared with drug response prediction, predicting surgical or interventional treatment response relies more heavily on assessing organ reserve, lesion reversibility, and post-procedural remodeling potential. Whilst conventional preoperative assessments can ascertain a patient's suitability for intervention based on symptoms, anatomical conditions, and clinical experience, they possess limited capacity to quantify the extent of postoperative benefit, the risk of complications, and the optimal timing. ML combined with multi-omics has the potential to identify patients most likely to benefit preoperatively, thereby optimizing surgical indications and the timing of intervention.

To illustrate this point, we may consider the challenge in predicting the response to surgical or interventional treatment using left ventricular assist device (LVAD) therapy. However, traditional preoperative assessments rely heavily on clinical and structural information, making it difficult to identify patients with the potential for recovery. To address this issue, Amrute et al. identified that the downregulation of RUNX1 regulatory networks in macrophages and fibroblasts was associated with postoperative functional recovery in LVAD patients. Furthermore, they constructed a random forest classifier and a Keras neural network model using preoperative RUNX1 target gene expression profiles; both models achieved AUCs exceeding 0.79, demonstrating their utility in predicting functional recovery (62). The present study translates “preoperative molecular status” into “probability of postoperative functional recovery” from a single-cell transcriptomic perspective. In the context of AS, conventional preoperative planning faces significant challenges in integrating patient-specific geometric, hemodynamic, and biomechanical data. To overcome this limitation, AI-driven multimodal CT modeling frameworks can now automatically generate high-fidelity patient-specific models. By integrating fluid-structure interaction and soft robotics models, these frameworks reconstruct critical hemodynamic features approximately 100 times faster than conventional workflows, thereby providing a more refined quantitative basis for personalized treatment planning, preoperative assessment, and interventional decision-making (43). These findings suggest that the value of predicting surgical response lies not merely in determining surgical feasibility, but in quantitatively estimating postoperative benefit from preoperative molecular and imaging data. Representative studies using ML and multi-omics to predict CVD risk and therapy response are summarized in Table 2.

**Table 2:** Summary of representative studies using machine learning and multi-omics for cardiovascular risk and therapy response prediction.

Task	Condition	Omics/Modality	Input	ML Algorithm(s)	Main outcome	Sample size	Validation type	Best performance	Risk of bias^a^	Applicability concern^a^	Reference
Primary Prevention	Healthy people	Proteomics	4,963 plasma proteins	LASSO Cox Model	First ASCVD event	Discovery: 9,522 Ext Val: 4,018	Ext Val	C-index = 0.747	Unclear risk	Unclear concern	(51)
Primary Prevention	Healthy people	Radiomics	CTH	Vision Transformer	Incident CVD	Discovery: 27,990	Int Val	C-index = 0.82	Unclear risk	Unclear concern	(54)
Secondary Prevention	STEMI	Metabolomics	130 oxylipins	Random Forest	Recurrent MACE	Discovery: 645 Ext Val: 401	Ext Val	Accuracy = 0.915	Unclear risk	Unclear concern	(55)
Secondary Prevention	CAD	Radiomics	CCTA	AI-QCT + Cox Model	All-cause mortality and nonfatal myocardial infarction	Discovery: 2,404	Int Val	AUC = 0.729-0.791	Unclear risk	Unclear concern	(56)
Short-term Prediction	AMI	Metabolomics	23 classes of metabolites	Random Forest	MACCE	Discovery: 3,223	Int Val	AUC = 0.715	High risk	Unclear concern	(58)
High-risk Population Management	Cancer Survivors	Proteomics	2,923 proteins	Random Forest	Major CVDs	Discovery: 4,225	Int Val	AUC = 0.646-0.665	High risk	Unclear concern	(59)
High-risk Population Management	CKD	Proteomics	4,638 proteins	Elastic-net + Cox Model	Incident heart failure	Discovery: 2,906 Ext Val: 1,136	Ext Val	C-index=0.79	Unclear risk	Unclear concern	(60)
Drug Response Prediction	Suspected CAD	Radiomics	CCTA	AI-QCT + Cox Model	Lipid-lowering benefit by PAV	Discovery: 2,269	Int Val	HR = 0.57 (PAV ≥ 5%) HR = 0.90 (PAV < 5%)	High risk	Unclear concern	(61)
Drug Response Prediction	Suspected CAD	Radiomics	CCTA	AI-CPA	Medication adjustment	Discovery: 972	Int Val	51.3% of patients had a management change	High risk	Unclear concern	(89)
Surgical Response Prediction	End-stage heart failure	Single-cell transcriptomics	7,081 snRNA-seq profiles from left ventricular specimens (RUNX1 target genes)	Neural Network, Random Forest	LVAD functional recovery	Discovery: 27	Ext Val	AUC = 0.945	/	/	(62)

CVD, cardiovascular disease; Major CVDs, heart failure, atrial fibrillation, myocardial infarction, angina, peripheral vascular disease, and stroke; LASSO, least absolute shrinkage and selection operator; AI-CPA, artificial intelligence-based coronary plaque analysis; AI-QCT, artificial intelligence-guided quantitative computed tomography; ASCVD, atherosclerotic cardiovascular disease; Ext Val, external validation; Int Val, internal validation; CTH, computed tomography of the head; STEMI, ST-elevation myocardial infarction; MACE, major adverse cardiovascular events; CAD, coronary artery disease; CCTA, coronary computed tomography angiography; AMI, acute myocardial infarction; MACCE, major adverse cardiac and cerebrovascular events; CKD, chronic kidney disease, PAV, percent atheroma volume, SnRNA, single-nucleus RNA; LVAD, left ventricular assist device.

a Risk of bias and applicability concerns were assessed using author-adapted criteria informed by the PROBAST framework. Detailed assessment criteria are provided in Supplementary Tables S1 and S2.

## 6 Challenges and perspectives

Multi-omics and ML have shown considerable promise in CVD diagnosis, risk assessment, and prediction of pharmacological and procedural outcomes. By integrating molecular, clinical, and imaging data, these technologies may support more individualized decision-making and improve the efficiency of cardiovascular care. Their broader value, however, depends on whether they can deliver non-invasive, comprehensive, reproducible, and clinically actionable assessments rather than merely achieve high performance in retrospective datasets.

Key priorities for clinical translation include improving omics data quality, developing robust strategies for multi-omics integration, incorporating emerging technologies such as spatial omics and generative artificial intelligence, strengthening model evaluation and oversight, and establishing reliable pathways for implementation in clinical decision-support systems.

### 6.1 Omics quality assessment and optimization of multi-omics integration

At the data level, variable data quality, batch effects, and systematic differences between analytical platforms can limit model generalizability and compromise multi-omics integration. Rigorous quality control and harmonization are therefore essential.

Standardized reporting and data-sharing frameworks provide the foundation for cross-study comparison. The HUPO Proteomics Standards Initiative continues to update data formats, controlled vocabularies, and minimum reporting requirements, while metabolomics has developed tiered frameworks covering analytical methods and quality control (91, 92). Study protocols should standardize sample collection, storage, and transport and document reagent lots, instrument models, and software versions to ensure traceability. Nevertheless, adherence to reporting standards does not eliminate variability across platforms or laboratories. Differences in assay sensitivity and coverage, instrument drift, and preprocessing procedures can all introduce systematic error (93, 94). In addition to statistical batch correction, multicenter studies should use reference materials and predefined quality-control procedures to monitor and correct analytical variability. Reporting coefficients of variation, recovery rates, and intra- and inter-batch consistency would further facilitate transparent auditing of data quality (92).

Translating exploratory omics findings into clinically validated assays requires stable and calibratable testing systems with well-defined reference intervals (95). Cabrera-Fuentes et al. proposed a tiered validation framework for plasma proteomics in cardiovascular precision medicine that progresses from candidate discovery to orthogonal validation using targeted mass spectrometry or independent immunoassays, genetic prioritization through colocalization and sensitivity analyses, tissue-resolved functional validation, prospective clinical evaluation, and regulatory approval (96). This process should also include analytical performance assessment, evaluation of clinical utility and cost-effectiveness, and independent replication across platforms, laboratories, and populations. Longitudinal studies should quantify intra-individual biological variability and define the minimum clinically important change so that technical variation is not misinterpreted as disease progression.

Multi-omics integration must account for differences among modalities in scale, distribution, dimensionality, and missing-data patterns. Some studies continue to rely on relatively simple fusion strategies, such as feature concatenation or aggregation of model outputs, which may fail to capture nonlinear interactions among omics layers. More recent approaches, including shared latent representations, variational autoencoders, and graph-based embeddings, can learn cross-omics features while retaining modality-specific information and may facilitate the alignment of unpaired data or the inference of missing modalities (97, 98). However, increased model complexity can heighten the risk of overfitting and reduce interpretability. Accordingly, studies should include single-omics benchmarks, ablation analyses, and independent external validation to determine the contribution and incremental value of each modality.

### 6.2 Expansion of the omics technologies

In terms of omics data sources, novel omics platforms such as spatial omics, modification omics, and circulating free nucleic acids are expected to expand into new biological dimensions, providing deeper insights that reflect the true nature of diseases.

Spatial omics, which includes spatial proteomics and spatial transcriptomics, preserves *in situ* positional information within tissues, thereby addressing the shortcoming of single-cell omics in losing spatial microenvironment information (99). Leveraging high-resolution platforms such as MERFISH and Stereo-seq enables analysis of the spatial distribution and intercellular interactions of different cell subsets within lesions of diseases such as cardiomyopathy and AS. For example, a related study using spatial transcriptomics analysis found that high expression of Trem2 macrophages in the infarct margin contributes to cardiac repair and the fibrosis process through *in situ* signaling (100). When combined with machine learning analysis, these approaches may reveal the spatiotemporal evolution of diseases, providing more accurate temporal predictions and classification of evolutionary patterns for the prognosis of CVDs (101). However, spatial omics relies on tissue biopsies rather than circulating bodily fluids such as plasma, presenting practical challenges such as sample collection and testing costs; the actual benefits of its clinical translation must also be carefully evaluated (99).

Epigenomics, represented by DNA methylation signals, serves as a more stable, noninvasive biomarker compared to the highly variable transcriptomic and proteomic markers, and holds significant potential for clinical translation. Furthermore, compared with proteomic and metabolomic markers that are more susceptible to temporal fluctuations caused by physiological status, environmental factors, and disease progression, this marker exhibits greater stability and reproducibility, making it a more reliable biomarker for long-term risk assessment (102–105). Related studies have demonstrated that it can reveal associations between mechanical stress-mediated early atherosclerotic progression, aneurysm risk, and specific DNA methylation patterns (103, 105). Research in the field of oncology has utilized ML to integrate multi-omics data on lactylation modifications, constructing a three-gene prognostic signature to stratify ovarian cancer prognosis and immunotherapy response. This study also validated the oncogenic function of NDUFS6, a gene associated with core modifications, and screened for targeted small molecules, providing a model for modificationomics-guided precision anticancer therapy (106). In the future, modificationomics can be integrated with other omics systems, such as transcriptomics and metabolomics, to facilitate the development of precision medicine through a more systematic analysis of CVDs (102).

Circulating free nucleic acids (cfDNA, cfRNA) are free nucleic acid fragments released into the bloodstream following cell apoptosis or necrosis; they serve as noninvasive biomarkers with multiple advantages, including repeatable dynamic detection, damage tracing, and early warning. Furthermore, compared with proteomic and metabolomic markers that are more susceptible to temporal fluctuations caused by physiological status, environmental factors, and disease progression, this marker exhibits greater stability and reproducibility, making it a more reliable biomarker for long-term risk assessment. Studies have shown its value in early screening and prognostic assessment for diseases such as HF, PAH, and myocarditis (107–110). For example, in a PAH cohort, total peripheral cfDNA levels were significantly positively correlated with disease severity; methylation deconvolution enabled the tracing of damaged cell types such as cardiomyocytes and vascular endothelial cells, and cfDNA levels independently predicted long-term adverse survival outcomes in patients (111). In the field of oncology, successful paradigms combining circulating free nucleic acids (cfDNA, cfRNA) with ML and DL have already been established. For example, analyzing cfDNA methylation using the Transformer-based MethyIBERT model enables the precise diagnosis of early-stage ovarian cancer, providing a mature technical reference for the development of cardiovascular cfDNA/cfRNA diagnostic and predictive models (112–114). Circulating free nucleic acids hold broad research prospects; however, it should be noted that the concentration of free nucleic acids in plasma is low, and pre-sequencing processing is prone to introducing technical biases, necessitating the standardization and regulation of relevant procedures (115, 116).

### 6.3 Generative AI and generative omics

Generative AI refers to a class of artificial intelligence technologies that generate new content with a certain degree of realism by learning statistical patterns and latent structures from existing data. Typical technologies include Transformer models, generative adversarial networks (GANs), autoencoders, and diffusion models. Large Language Models (LLMs) are a key component of generative AI. They primarily utilize the Transformer architecture with self-attention mechanisms to understand long-range semantic relationships and have potential applications in clinical decision-making, biomedical knowledge integration, and multi-omics research within the cardiovascular field. For example, LLMs can be used to analyze population-based health examination data to build whole-body and cardiac-specific aging prediction models, as well as to generate risk prediction reports for hundreds of diseases in bulk to aid clinical decision-making, thereby providing research tools and paradigms for the comprehensive management of CVDs (117, 118). LLMs can also integrate vast amounts of biomedical knowledge—such as medical papers, clinical guidelines, and databases—to form knowledge networks, understand human instructions, and generate expert answers. Relevant models include BioBERT, ClinicalBERT, GatorTron, and Me-LLaMA (119). However, when using such models, one must be vigilant regarding issues of credibility and hallucination. In multi-omics research, agents such as MEDEA have already been developed to perform end-to-end omics analysis and have been tested and validated in three scenarios: target discovery, synthetic lethality inference, and immunotherapy response prediction (120). By comprehensively applying relevant models, it is possible to achieve the organic integration of existing medical knowledge with omics information, perform end-to-end automated analysis, and generate cardiovascular diagnosis and treatment recommendations.

Generative omics refers to an emerging technological direction that utilizes generative AI models to generate, augment, or simulate multi-omics data. By leveraging generative omics models, it is possible to synthesize a large number of omics profiles that resemble real data but are not simple replicas, thereby addressing existing challenges or needs such as data scarcity and data accessibility. For example, in oncology, the OncoGAN model utilizes generative adversarial networks (GANs) and variational autoencoders (VAEs) to learn from real tumor genomes and generate simulated tumor genomes containing known mutation information. Since these genomes are not linked to patient identities, they offer a solution to privacy barriers in data sharing. Generative omics can also provide tools and methods to “fill in the gaps” for missing omics modalities in multi-omics integration (121). For example, the AURORA framework can generate virtual omics profiles covering seven modalities—including the transcriptome and metabolome—using only facial photographs or routine blood test reports, and has been validated on a dataset of over 420,000 individuals (122). Finally, generative omics can also simulate and visualize dynamic omics changes following drug treatment. Although it is currently used primarily for drug target screening, it can also be applied to predict the efficacy of interventions by inferring core omics features from clinical outcomes (123). In summary, generative omics leverages powerful computational capabilities to enable large-scale multi-omics research to be conducted more cost-effectively and efficiently.

### 6.4 Transfer learning and foundation models

Supervised training relies on large-scale, high-quality labeled data; in the field of omics, acquiring such data is costly. Transfer learning and foundational models offer important future strategies to address data constraints in this field. Transfer learning enables models to learn general underlying patterns by pre-training on broad, relevant data, after which they are fine-tuned on limited medically annotated data to adapt to specific clinical tasks, thereby reducing costs, improving performance, or enabling cross-dataset applicability (124, 125). This strategy is currently used with imaging data and may be applicable in the future for multi-omics integration to explore interactions between omics disciplines (126). Foundational models are powerful tools for transfer learning; by learning extensively from massive amounts of data, they can be adapted to numerous tasks through fine-tuning. Single-modality foundational models, such as scGPT—a foundational model for single-cell omics analysis—are pre-trained on over 33 million cells and can subsequently be fine-tuned for tasks such as cell-type annotation, multi-batch integration, and multi-omics integration (127). Multimodal foundation models, on the other hand, can integrate single-modal foundation models within a unified framework. For example, HONeYBEE comprises foundation models for five key modalities—including clinical, imaging, and molecular data—enabling the integration of multimodal data and the execution of tasks such as end-to-end cancer subtype classification (128). Additionally, some foundation models are capable of broadly learning representations and transferring them to diverse medical applications. For instance, the Cardiac Sensing Foundation Model (CSFM), pre-trained on clinical data—including electrocardiograms—from 1.7 million individuals, can address various clinical scenarios such as CVD diagnosis and outcome prediction (129). Leveraging transfer learning and foundation models to fuse multimodal data and obtain more robust (stable) results is a key developmental direction for cardiovascular multi-omics integrated with ML. However, the risks associated with foundation models include implicit architectures and single points of failure—that is, because the field tends to rely on a small number of foundation models, defects in upstream models may be inherited by downstream applications or lead to data privacy breaches, among other issues (130). Through subsequent interpretability enhancements and the establishment of a security framework, this approach holds significant translational potential.

### 6.5 Model overoptimism and quality assessment

Although many machine-learning studies report area under the receiver operating characteristic curve values above 0.90, such estimates may be substantially overoptimistic and should not be equated with clinical utility (131). Most studies are based on small, single-center, retrospective cohorts with high-dimensional predictors. Complex models may learn cohort-specific noise, resulting in poor generalizability, while the absence of independent validation across institutions and populations obscures their performance in routine practice (132).

Insufficient reporting further complicates assessment. Many studies do not provide adequate information about the data used or the model-development process, making it difficult to determine whether data leakage occurred, such as the direct or indirect use of validation or test-set information during training (133). In prognostic studies, simply excluding competing events, such as non-cardiovascular death, may overestimate the cumulative incidence of the target outcome and inflate apparent model performance (134). Retrospective designs are also vulnerable to selection bias arising from case-control sampling, exclusion of participants with incomplete data, class imbalance, and restricted disease spectra. Differences in genetic background, socioeconomic conditions, treatment pathways, and diagnostic equipment may further limit transportability across populations, regions, and healthcare settings (132).

Model assessment should therefore extend beyond discrimination. Classification models should report sensitivity, specificity, F1 scores, and other clinically relevant measures, whereas prognostic and survival models should additionally assess calibration-in-the-large, calibration slopes, Brier scores, and time-dependent performance. Decision-curve analysis and net benefit should be used to determine whether a model improves clinical decision-making at relevant thresholds (135). Tools such as the Prediction model Risk Of Bias Assessment Tool (PROBAST) can support structured evaluation of risk of bias, applicability, and methodological quality across study populations, data sources, predictors, and validation procedures (136).

Diagnostic and prognostic models address different questions: the former estimate the presence of disease, whereas the latter predict future events. They should therefore not be conflated with respect to study design, outcome definitions, or validation methods. Internal and external validation must remain strictly separate from model development to prevent leakage and biased performance estimates. For models intended for clinical use, prospective evaluation is particularly important because performance and utility observed retrospectively may not persist after deployment. Continuous validation across institutions, devices, and populations should monitor performance drift, fairness, and unintended consequences. Such an approach is necessary to narrow the gap between promising research results and reliable clinical tools (137).

### 6.6 Implementation of clinical decision-support systems

The clinical value of machine-learning and multi-omics models ultimately depends on their integration into real-world workflows and their ability to generate actionable decisions. An American Heart Association (AHA) scientific statement emphasizes that adoption should not be based on predictive performance alone. Incremental clinical benefit, usability, scalability, generalizability, reproducibility, cost-effectiveness, and health equity should also be evaluated (138). Model inputs should connect to electronic health records, imaging platforms, and laboratory information systems through standardized interfaces. Outputs should clearly specify the target population, prediction horizon, uncertainty, influential features, and recommended actions. The DECIDE registry study, which translated quantitative coronary CT angiography findings into cardiovascular prevention and management recommendations and incorporated them into clinical pathways, provides an example of real-world implementation (89). In most settings, a human-machine partnership is preferable: AI can integrate complex data and provide decision support, while clinicians retain responsibility for the final decision, incorporating patient preferences, comorbidities, contraindications, and broader clinical context (139).

Implementation also requires appropriate training and operational safeguards. Clinicians should understand the model's intended population, calibration, common sources of bias, and procedures for managing abnormal or uncertain outputs. Healthcare institutions should establish mechanisms for manual review, overriding model recommendations, escalating faults, and reporting incidents. Decision-support systems should provide timely information linked to clear action pathways and minimize alert fatigue through tiered thresholds, consolidation of duplicate alerts, limits on alert frequency, display of triggering criteria, and monitoring of response and override rates (140, 141).

Regulatory and ethical requirements should reflect the intended use and risk profile of each tool. In the United States, omics test kits and analytical systems are generally regulated as *in vitro* diagnostic devices, whereas machine-learning software with an independent medical function may fall within the framework for software as a medical device (SaMD). Depending on the risk level, the relevant regulatory pathway should address analytical and clinical performance, quality systems, and post-market surveillance. For adaptive or continuously updated models, the scope of permissible modifications, requirements for revalidation, and procedures for version rollback should be defined in advance (142, 143). Responsibilities must also be clearly allocated. Developers should ensure algorithmic integrity and the quality of updates; healthcare institutions should conduct local validation and maintain deployment security; and clinicians should apply model outputs within individualized clinical decision-making. Comprehensive audit logs should be retained. Informed consent for multi-omics testing should address the scope of testing, secondary use of data, cross-institutional sharing, and the return of incidental findings. Genomic and proteomic data should be protected through data minimization, tiered authorization, de-identification, encryption, and access auditing, in accordance with the General Data Protection Regulation (GDPR), the Health Insurance Portability and Accountability Act (HIPAA), and applicable local requirements (144).

Cost and reimbursement will strongly influence long-term adoption. Research-grade testing typically costs approximately $100–500 for genomics, $200–1,000 for proteomics, and $100–500 per metabolomics sample; the total cost also includes sample logistics, repeated quality-control testing, data storage, algorithm licensing, expert interpretation, and confirmatory follow-up (145, 146). Cost-effectiveness cannot be defined by a universal price threshold. It depends on the baseline risk of the target population, testing frequency, the proportion of clinical decisions changed by the model, incremental costs and benefits, and the potential to avoid invasive procedures or adverse events. Some artificial intelligence-assisted coronary CT angiography services have entered the coding and reimbursement process through the Centers for Medicare & Medicaid Services, although coverage remains dependent on specific indications and policies (147). Future prospective implementation studies should quantify resource use, downstream healthcare costs, quality-adjusted life years, and equity-related outcomes to determine whether these technologies improve outcomes while promoting efficient resource allocation.

Overall, multi-omics and ML can connect molecular mechanisms with clinical decision-making in cardiovascular precision medicine. Their value, however, should not be judged by a single performance metric or by technological novelty alone. Reproducible assays, transparent and robust integration methods, rigorous external and prospective validation, and implementation systems that are auditable, affordable, and centered on shared decision-making are required for these approaches to progress from research tools to safe, effective, and equitable clinical practice.

## Glossary

AAS: acute aortic syndrome
ACS: acute coronary syndrome
AHA: american heart association
AI: artificial intelligence
AI-CPA: artificial intelligence-based coronary plaque analysis
AI-QCT: artificial intelligence-guided quantitative computed tomography
AMI: acute myocardial infarction
AS: aortic stenosis
ASCVD: atherosclerotic cardiovascular disease
ATTR-CA: transthyretin-related cardiac amyloidosis
AUC: area under the curve
CA: cardiac amyloidosis
CAC: coronary artery calcium
CAD: coronary artery disease
CAD-RADS: coronary artery disease reporting and data system
CCTA: coronary computed tomography angiography
cfDNA: circulating free DNA
cfRNA: circulating free RNA
CKD: chronic kidney disease
CMR: cardiovascular magnetic resonance
CNN: convolutional neural network
CT: computed tomography
CTA: computed tomography angiography
CTPA: computed tomography pulmonary angiography
CVDs: cardiovascular diseases
DL: deep learning
DT: decision tree
EDV: end-diastolic volume
EF: ejection fraction
EHT: endocrine hypertension
ESV: end-systolic volume
GAN: generative adversarial network
GDPR: general data protection regulation
HCM: hypertrophic cardiomyopathy
HF: heart failure
HFpEF: heart failure with preserved ejection fraction
HIPAA: health insurance portability and accountability act
ICA: invasive coronary angiography
LASSO: least absolute shrinkage and selection operator
LDL-C: low-density lipoprotein cholesterol
LGE: late gadolinium enhancement
LLM/LLMs: large language model(s)
LR: logistic regression
LSTM: long short-term memory
LV mass: left ventricular mass
LVAD: left ventricular assist device
LVEF: left ventricular ejection fraction
LVOT-VTI: left ventricular outflow tract velocity-time integral
MACCE: major adverse cardiac and cerebrovascular events
MACE: major adverse cardiovascular events
MI: myocardial infarction
ML: machine learning
mPAP: mean pulmonary artery pressure
MRI: magnetic resonance imaging
NRI: net reclassification index
NSTEMI: non-ST-elevation myocardial infarction
NT-proBNP: N-Terminal pro-B-type natriuretic peptide
PAH: pulmonary arterial hypertension
PAV: percent atheroma volume
PCI: percutaneous coronary intervention
PET: positron emission tomography
PET/CT: positron emission tomography/computed tomography
PHT: primary hypertension
PROBAST: prediction model risk of bias assessment tool
RF: random forest
RNN: recurrent neural network
RUNX1: runt-related transcription factor 1
RVWT: right ventricular wall thickness
SHAP: shapley additive explanations
SISSO: sure independence screening and sparsifying operator
SaMD: software as a medical device
SNF: similarity network fusion
STEMI: ST-elevation myocardial infarction
SV: stroke volume
SVM: support vector machine
U-Net: U-shaped convolutional neural network
VAE/VAEs: variational autoencoder(s)
XGBoost: eXtreme gradient boosting
