# Supplementary material for: Machine learning and multi-omics technologies for precision cardiovascular medicine: advancing diagnosis, risk prediction, and therapeutic guidance
Source: Front Cardiovasc Med. 2026 Aug 13;13:1921962. doi: 10.3389/fcvm.2026.1921962 (PMC13471024; doi:10.3389/fcvm.2026.1921962)
Supplement: Supplementary file 1 [file Table1.docx]

**Table S1 PROBAST evaluation result**

| Reference | Risk of Bias | | | | Applicability | | | Overall | |
| --- | --- | --- | --- | --- | --- | --- | --- | --- | --- |
|  | Participants | Predictors | Outcome | Analysis | Participants | Predictors | Outcome | Risk of bias | Applicability |
| Lin et al., 2022 | + | + | ? | + | + | + | + | ? | + |
| Zeng et al., 2025 | + | + | ? | + | + | + | + | ? | + |
| Shad et al., 2026 | + | + | ? | + | + | + | + | ? | + |
| Versnjak et al., 2025 | + | + | + | ? | ? | + | + | ? | ? |
| Mori et al., 2025 | + | + | + | + | + | + | + | + | + |
| Hong et al., 2025 | - | ? | + | - | ? | + | + | - | ? |
| Slivnick et al., 2025 | + | + | ? | + | + | + | + | ? | + |
| Hu et al., 2025 | + | + | ? | + | + | + | + | ? | + |
| Gu et al., 2025 | - | + | ? | - | ? | + | + | - | ? |
| Vrudhula et al., 2024 | + | + | ? | + | + | + | + | ? | + |
| Helgason et al., 2023 | + | + | + | ? | ? | + | + | ? | ? |
| Zhang et al., 2026 | + | + | + | ? | ? | + | + | ? | ? |
| Du et al., 2025 | + | + | + | ? | ? | + | + | ? | ? |
| Dahdal et al., 2025 | + | + | + | ? | ? | + | + | ? | ? |
| Xiu et al., 2026 | - | + | + | - | ? | + | + | - | ? |
| Wang et al., 2026 | - | + | + | - | ? | + | + | - | ? |
| Dubin et al., 2024 | + | + | + | ? | ? | + | + | ? | ? |
| Maaniitty et al., 2026 | - | + | + | - | ? | + | + | - | ? |
| Rinehart et al., 2026 | - | + | ? | - | ? | + | + | - | ? |
| Amrute et al., 2023 | / | / | / | / | / | / | / | / | / |

**Table S2 Assessment criteria of PROBAST evaluation result**

|  | + |  | ? | / |
| --- | --- | --- | --- | --- |
| Risk of Bias Assessment | | | |  |
| Participants | Adequate participant selection (e.g., consecutive enrollment, appropriate exclusion criteria) | High risk (e.g., case-control design, restrictive selection) | Unclear reporting of selection methods |  |
| Predictors | Standardized and reproducible measurement (e.g., validated omics pipeline, established imaging protocol) | Non-standardized assessment without quality control | Insufficient details on measurement procedures |  |
| Outcome | Blinded and objective outcome ascertainment using established diagnostic criteria | Subjective or unblinded outcome determination | Unclear outcome definition or assessment procedure |  |
| Analysis | Appropriate analytic strategy (e.g., adequate sample size, proper handling of overfitting, external validation when applicable) | Major methodological flaws (e.g., severe overfitting, data leakage, no validation) | Insufficient reporting of statistical methods |  |
| Applicability | | | |  |
| Participants | Representative of the target clinical population (e.g., multi-center, broad demographics) | Narrow or highly selected population limiting generalizability | Population characteristics insufficiently described |  |
| Predictors | Widely available in routine clinical settings (e.g., standard imaging, common laboratory tests) | Requires specialized or rare equipment not broadly accessible | Availability in routine practice unclear |  |
| Outcome | Clinically meaningful endpoint (e.g., confirmed diagnosis, major adverse cardiovascular events) | Surrogate endpoint with unclear clinical relevance | Clinical importance of outcome uncertain |  |
| Overall Assessment | | | |  |
| Risk of Bias | Low risk (all domains rated '+') | High risk (one or more '−') | Unclear risk (at least one '?' without any '−') |  |
| Generality | High generalizability (all domains rated '+') | Low generalizability (one or more '−') | Unclear (at least one '?' without any '−') | Not applicable (insufficient information, single-case study settings, or non-applicable PROBAST criteria). |

1.	Lin A, Manral N, McElhinney P, Killekar A, Matsumoto H, Kwiecinski J, et al. Deep learning-enabled coronary CT angiography for plaque and stenosis quantification and cardiac risk prediction: an international multicentre study. The Lancet Digital health. 2022;4(4):e256-e65.

2.	Zeng Y, Ling G, Zhang H, Cao W, Zheng X, Deng X, et al. Artificial intelligence-driven multivariate integration for pulmonary arterial pressure prediction in pulmonary hypertension. NPJ digital medicine. 2025;9(1):56.

3.	Shad R, Zakka C, Kaur D, Mathur M, Fong R, Cho J, et al. A generalizable deep learning system for cardiac MRI. Nature biomedical engineering. 2026.

4.	Versnjak J, Kuehne T, Fahjen P, Jovanovic N, Löber U, Schiattarella GG, et al. Deep phenotyping of heart failure with preserved ejection fraction through multi-omics integration. Eur J Heart Fail. 2025;27(12):3243-59.

5.	Mori S, Montobbio N, Sormani MP, Campi C, Mazzoni C, Argirò A, et al. Echocardiographic Tissue Characterization Using Radiomics in Patients With Transthyretin-Related Cardiac Amyloidosis. JACC Advances. 2025;4(6 Pt 1):101755.

6.	Hong Z, Spielvogel CP, Xue S, Calabretta R, Jiang Z, Yu J, et al. Enhanced diagnostic and prognostic assessment of cardiac amyloidosis using combined (11)C-PiB PET/CT and (99m)Tc-DPD scintigraphy. European journal of nuclear medicine and molecular imaging. 2025;52(9):3321-32.

7.	Slivnick JA, Hawkes W, Oliveira J, Woodward G, Akerman A, Gomez A, et al. Cardiac amyloidosis detection from a single echocardiographic video clip: a novel artificial intelligence-based screening tool. Eur Heart J. 2025;46(40):4090-101.

8.	Hu Y, Xiang Y, Zhou YJ, He Y, Lang D, Yang S, et al. AI-based diagnosis of acute aortic syndrome from noncontrast CT. Nature medicine. 2025;31(11):3832-44.

9.	Gu AN, Vaseli H, Tsang MY, Wu V, Ahmadi Amiri SN, Kondori N, et al. ProtoASNet: Comprehensive evaluation and enhanced performance with uncertainty estimation for aortic stenosis classification in echocardiography. Medical image analysis. 2025;103:103600.

10.	Vrudhula A, Duffy G, Vukadinovic M, Liang D, Cheng S, Ouyang D. High-Throughput Deep Learning Detection of Mitral Regurgitation. Circulation. 2024;150(12):923-33.

11.	Helgason H, Eiriksdottir T, Ulfarsson MO, Choudhary A, Lund SH, Ivarsdottir EV, et al. Evaluation of Large-Scale Proteomics for Prediction of Cardiovascular Events. Jama. 2023;330(8):725-35.

12.	Zhang X, Acosta JN, Dogra S, Silva EN, Basu S, Krumholz HM, et al. Opportunistic Cardiovascular Risk Assessment Using Routine Head CT in the Emergency Department. Journal of the American College of Cardiology. 2026.

13.	Du Z, Lu Y, Ma Y, Yang Y, Luo W, Liu S, et al. The prognostic and therapeutic significance of polyunsaturated fatty acid-derived oxylipins in ST-segment elevation myocardial infarction. iMeta. 2025;4(1):e266.

14.	Dahdal J, Jukema RA, Maaniitty T, Nurmohamed NS, Raijmakers PG, Hoek R, et al. CCTA-Derived coronary plaque burden offers enhanced prognostic value over CAC scoring in suspected CAD patients. European heart journal Cardiovascular Imaging. 2025;26(6):945-54.

15.	Xiu L, Liu Y, Wu P, Wang M, Cui H, Zhao J, et al. Tryptophan metabolites and stroke risk after acute myocardial infarction in patients with and without metabolic syndrome: insights from a MACCE-based cohort. Cardiovascular diabetology. 2026;25(1).

16.	Wang P, Peng Y, Liu F, Sheng C, Chen K, Song F. Cardiovascular risk assessment characterized by proteomics in cancer survivors. Communications medicine. 2026.

17.	Dubin RF, Deo R, Ren Y, Wang J, Pico AR, Mychaleckyj JC, et al. Incident heart failure in chronic kidney disease: proteomics informs biology and risk stratification. Eur Heart J. 2024;45(30):2752-67.

18.	Maaniitty T, Bär S, Bax JJ, Knuuti J, Saraste A. Quantitative Coronary Atherosclerotic Plaque Burden From CCTA and the Benefit From Lipid-Lowering Medication. Circulation Cardiovascular imaging. 2026;19(4):e018840.

19.	Rinehart S, Blankstein R, McCarthy CP, Januzzi JL, Scherer M, O'Neal WT, et al. Guiding Preventive Care Strategies for Patients With Atherosclerotic Plaque on Coronary CTA: Primary Outcomes of the DECIDE Registry. JACC Cardiovascular imaging. 2026.

20.	Amrute JM, Lai L, Ma P, Koenig AL, Kamimoto K, Bredemeyer A, et al. Defining cardiac functional recovery in end-stage heart failure at single-cell resolution. Nature cardiovascular research. 2023;2(4):399-416.
